# L’éléphantiasis vulvo-clitoridien: à propos d'un nouveau cas

**DOI:** 10.11604/pamj.2015.22.133.7933

**Published:** 2015-10-13

**Authors:** Babacar Sine, Ndeye Aissatou Bagayogo, Amath Thiam, Alioune Sarr, Yaya Sow, Boubacar Fall, Abdou Razak Hamidou Zakou, Samba Thiapato Faye, Babacar Diao, Papa Ahmed Fall, Alain khassim Ndoye, Mamadou Ba

**Affiliations:** 1 Service d'Urologie-Andrologie de l'Hôpital Aristide Le Dantec de Dakar, Sénégal

**Keywords:** Eléphantiasis, filariose, vulvo-clitoridien, Elephantiasis, filariasis, vulvo-clitoral

## Abstract

L’éléphantiasis vulvo-clitoridien d'origine filarienne est une affection très rare. Nous rapportons un nouveau cas chez une femme de 33 ans suivie dans un service de Maladies Infectieuses pour filariose lymphatique. Elle avait une masse vulvo-clitoridienne qui évoluait depuis plus de 10 ans. Une résection clitoridienne et une plastie vulvaire a été réalisée. Les résultats fonctionnels et esthétiques étaient satisfaisants.

## Introduction

L’éléphantiasis des organes génitaux externes est caractérisé par une lymphangite chronique avec hypertrophie des organes génitaux externes. C'est une affection rare d’étiologie variée dont la plus fréquente est la filariose. C'est une affection qui peut avoir un retentissement sexuel, professionnel, sportif, et pose esthétique et voire psychologique [1]. Il est assez fréquent chez l'homme mais seulement deux cas ont été décrits chez la femme. Nous rapportons un nouveau cas d’éléphantiasis des OGE chez une femme dont nous décrivons la présentation clinique, le traitement et le résultat.

## Patient et observation

**Figure 1:**
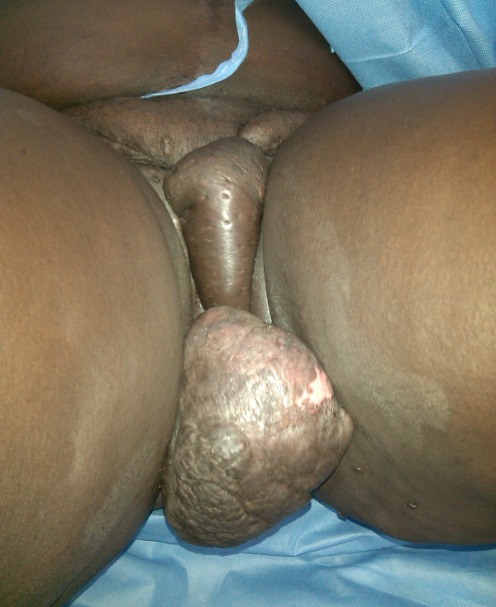
Masse clitoridienne appendue sur la racine du clitoris et masse de la grande lèvre gauche remontant jusqu’à la partie gauche du pubis

**Figure 2:**
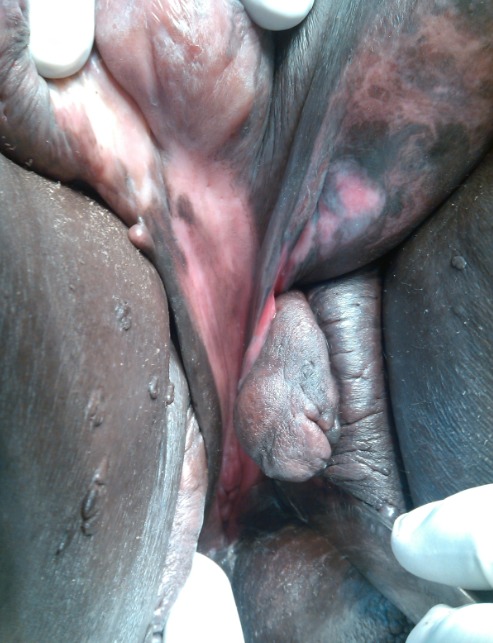
La surélévation de la masse clitoridienne laisse apparaître un vagin et un méat urétral sains

**Figure 3:**
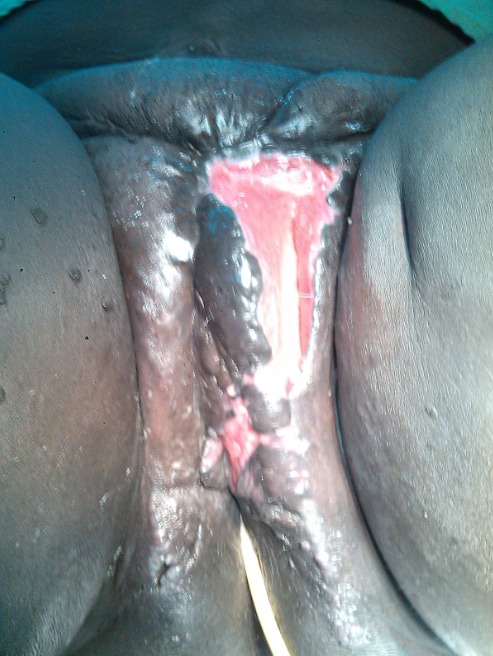
Lâchage des sutures 9 jours après l'opération

**Figure 4:**
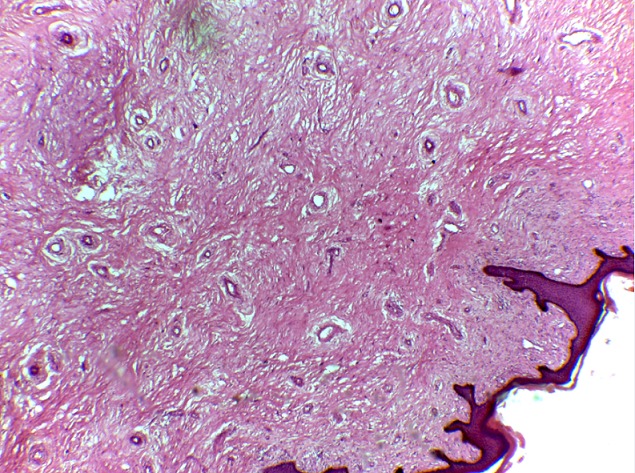
L'examen anatomo-pathologique mettant en évidence un épiderme acanthosique et hyperkératosique; le derme sous-jacent présentait une hyperplasie des fibrolastes et des léomiocytes; il existait des vaisseaux ectasiques à endothélium aplati et à paroi fibreuse entourés d'un infiltrat lymphocytaire sans véritable vascularisation

**Figure 5:**
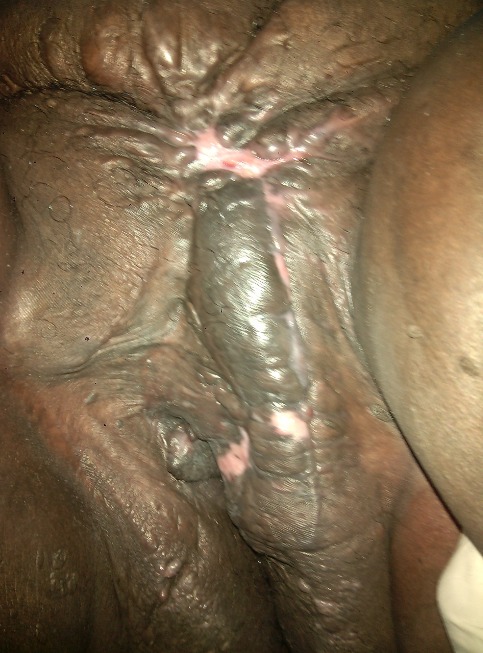
Aspect final 1 mois après l'opération

RN âgée de 33 ans avait été adressée dans notre service pour la prise en charge d'un éléphantiasis des OGE évoluant depuis plus de 10 ans. L'interrogatoire a révélé qu'il s'agissait d'une augmentation de volume du membre supérieur droit puis du membre inférieur homolatéral qui avait nécessité une consultation au service des maladies infectieuses. Elle a ainsi pris plusieurs doses de Diethylcarbamazine (DEC) sans amélioration. Par la suite une augmentation de volume des OGE est apparue progressivement gênant la marche, l'accouchement et l'activité sexuelle. Dans ses antécédents, il existait deux grossesses et deux accouchements par césarienne. Elle était divorcée depuis 5 ans et elle était indigente. L'examen à l'admission avait mis en évidence un bon état général, un trouble de la marche, une obésité avec un Indice de Masse Corporelle (IMC) à 42,85, une hypertrophie du membre supérieur droit épargnant la main et du membre inférieur homolatéral avec pachydermie. Elle avait des cicatrices hypogastriques disgracieuses. Au niveau des OGE, il existait une hypertrophie du gland du clitoris maintenue par le frein et ulcérée par endroit, une hypertrophie de la grande lèvre gauche remontant jusqu’à la partie gauche du pubis. Le méat urétral et le vagin étaient libres (Figure 1, Figure 2). Ainsi, le diagnostic d’éléphantiasis des OGE a été retenu. La patiente a été opérée le 15 juin 2015 sous rachianesthésie. L'opération a consisté d'abord en une résection de la masse clitoridienne par section de la racine du clitoris Et ensuite une plastie vulvaire avec une incision en quartier d'orange de part et d'autre de la masse de la grande lèvre gauche du bord inférieur de la vulve jusqu'au pubis emportant la masse. La dissection du plan était progressive avec une hémostase progressive aux fils Vicryl 2/0 parfois au bistouri électrique. Un rapprochement sous cutané a été fait en deux plans par des points en « U » au Vicryl 3/0 puis la fermeture cutanée avec des points de Blaire Donatti au Vicryl 2/0. Une sonde transurétrale a été mise en place. Au 9ème jour postopératoire elle a eu une suppuration pariétale avec lâchage des sutures (Figure 3). Une reprise des sutures a été faite 15 jours plus tard sous anesthésie locorégionale. L'examen anatomo-pathologique avait mis en évidence un épiderme acanthosique et hyperkératosique. Le derme sous-jacent présentait une hyperplasie des fibrolastes et des léomiocytes. Il existait des vaisseaux ectasiques à endothélium aplati et à paroi fibreuse entourés d'un infiltrat lymphocytaire sans véritable vascularisation (Figure 4). Avec un recul de un mois, les résultats esthétiques et fonctionnels sont satisfaisants (Figure 5).

## Discussion

La filariose lymphatique est une affection fréquente dans la zone intertropicale. L’éléphantiasis peut intéresser les membres supérieurs, inférieurs mais également les organes génitaux externes. Ceci est lié à l'obstruction des vaisseaux lymphatiques par les vers adultes ou par inflammation puis fibrose ou par compression externe [2, 3]; Pour l’éléphantiasis des OGE, les hommes sont plus touchés que les femmes [4]. L′éléphantiasis de la vulve touche environ 1-2% des patients ayant un éléphantiasis filarien [5]. L’étiologie de l’éléphantiasis vulvaire peut être infectieuse (filariose, tuberculose, syphylis, donavanose, onchocerchose), radique, iatrogène (après chirurgie pelvienne) ou idiopathique [6]. Chez notre patiente, l’éléphantiasis est dû à la filariose lymphatique. C'est pourquoi elle a été suivie dans un service de maladies infectieuses où elle révèle avoir pris plusieurs doses de DEC. L’éléphantiasis clitoridien est souvent associé et la localisation vulvaire gauche a été rapportée dans les 2 cas d’éléphantiasis filarien publiés [7, 8]. La découverte d'un éléphantiasis vulvo-clitoridienne pose un problème esthétique et fonctionnel qui impose la reconstruction plastique. Le but du traitement qui est chirurgical à ce stade est de corriger la dysmorphie. Pour cela, la résection des tissus éléphantiasiques suivie d'une plastie constitue le traitement de référence [9, 10]. L’éléphantiasis vulvo-clitoridienne est tellement rare qu'il n'existe pas de traitement bien codifié sur le plan chirurgical comme l’éléphantiasis des OGE chez l'homme. Les cas publiés n'ont pas insisté sur les techniques de la chirurgie de reconstruction [5, 7, 8]. Les résultats sont souvent satisfaisants. Cependant il peut exister des récidives surtout si la résection n'est pas complète [7]. Notre patient a eu une bonne évolution. La cicatrisation a été retardée par une suppuration pariétale avec lâchage de suture favorisée par la macération du fait de l'obésité morbide que la patiente avait. L'aspect des OGE était satisfaisant sur le plan fonctionnel et esthétique après un recul d'un mois.

## Conclusion

L’éléphantiasis des OGE est une affection très rare chez la femme. Le retentissement fonctionnel, psychologique et sexuel est important. C'est ce qui impose souvent le traitement qui n'est que chirurgicale à ce stade dans la majorité des cas.
